# Does arch length preservation in mixed dentition children affect mandibular second permanent molar eruption? A systematic review and meta-analysis

**DOI:** 10.1186/s12903-021-01755-1

**Published:** 2021-08-11

**Authors:** Sivakumar Arunachalam, Indumathi Sivakumar, Jayakumar Jayaraman, Jitendra Sharan

**Affiliations:** 1 School of Dentistry, International Medical University, No.126, Jalan Jalil Perkasa 19, Bukit Jalil, Kuala Lumpur, Malaysia; 2 Faculty of Dentistry, SEGi University, Kuala Lumpur, Malaysia; 3 Department of Developmental Dentistry, University of Texas Health School of Dentistry, 7703 Floyd Curl Drive, San Antonio, TX 78229 USA; 4 Department of Dentistry, AIIMS, Bhubaneswar, India

**Keywords:** Leeway space, E- space, Arch length, Lip bumper, Lingual holding arch, Molar impaction, Eruption difficulties

## Abstract

**Background:**

Arch length preservation strategies utilize leeway space or E-space in the mixed dentition to resolve mild to moderate mandibular incisor crowding. The purpose of this systematic review of the literature was to analyze the effects of arch length preservation strategies in on mandibular second permanent molar eruption.

**Methods:**

A search for relevant articles published from inception until May 2020 was performed using PubMed/Medline, Cochrane databases, Clinicaltrials.gov, Google scholar and journal databases. Preferred Reporting Items for Systematic Reviews and Meta-Analyses (PRISMA) guidelines were adopted for the conduct of the systematic review. Using RevMan 5.3 software, the most pertinent data were extracted and pooled for quantitative analysis with 95% confidence intervals. Heterogeneity was analyzed by using Cochran Q test and I squared statistics.

**Results:**

A total of 5 studies involving 855 mixed dentition patients with arch length preservation therapy were included in the qualitative analysis. Pooled estimate of the data from two studies revealed 3.14 times higher odds of developing mandibular second molar eruption difficulty due to arch length preservation strategies using lingual holding arch (95% CI; OR 1.10–8.92). There was no heterogeneity found in the analysis. The certainty levels were graded as very low.

**Conclusions:**

This systematic review demonstrates that arch length preservation strategies pose a risk for development of mandibular second molar eruption disturbances, but the evidence was of very low quality.

*Registration number*: CRD42019116643.

## Background

Loss of mandibular arch length is an inevitable event during the transitional period, and it was estimated to be about 1.8 mm per side of the arch [1]. This phenomenon raised a reasonable question that if simple arch length preservation during the transition period could provide adequate space to manage crowding in the mixed dentition without any active intervention [2]. Accordingly, utilization of leeway space of Nance/E-space just prior to exfoliation of the mandibular second primary molar through arch length preservation strategies (ALPS) for the relief of mandibular anterior crowding has been suggested [2–4].

Several investigators consistently demonstrated the effectiveness of arch length preservation in preventing mesial migration of the permanent first molars [5–7]. A recent systematic review reported 5.1 mm resolution of mandibular incisor crowding with passive lower lingual arch therapy [8]. However, conflicting notion exists in the literature with regards to the early management of mandibular incisor crowding and resultant long-term dental health benefits [9, 10]. Further, clinical studies could not demonstrate long-term lower incisor positional stability through ALPS when compared to mixed dentition expansion protocols or extraction of premolars [11].

With the probability of successful early management of crowding using arch length preservation strategies, researchers attempted to explore further on the process of natural transitional mechanism in the dentition and its impediments, if any [12, 13]. They noted that it may not be prudent to manage the anterior arch discrepancy without creating a posterior arch discrepancy [12]. On a general note, Paulo and Betty demonstrated some risk of mandibular second permanent molar (M2) impaction in a sample of patients undergoing orthodontic treatment [14].

More recently, studies utilizing ALPS have reported an increase in the incidence of M2 eruption difficulties leading to impaction or ectopic eruption [15–19]. A reported incidence in the range of 4.7–14.5% was noted with lingual holding arch and 11.9–22% with lip bumper [15–19]. However, prevalence of impacted M2s in the general population ranged from 0.2 to 2.3% [20, 21]. The aim of this study was to systematically review the effects of ALPS in mixed dentition on mandibular second permanent molar eruption.

## Methods

### Protocol and registration

Guidelines from ‘Preferred Reporting Items for Systematic Reviews and Meta-Analyses (PRISMA)’ helped to report this review in concordance [22]. The review protocol was registered in PROSPERO International Prospective Register of Systematic Reviews (CRD42019116643).

### Eligibility criteria

The methodology included formulating review questions using a Population, Exposure, Comparison, Outcome, Study design (PECOS) framework (Table 1), constructing a search strategy, defining inclusion and exclusion criteria, locating studies, selecting studies, assessing study quality, extracting data, and forming an evidence table prior to interpretation. The research question formulated for this study was as follows: Does arch length preservation strategies in the mixed dentition affect mandibular second permanent molar eruption?

**Table 1:** Population, exposure, comparison, outcome, study design (PECOS) framework

Population	Children with mixed dentition
Exposure	Orthodontic treatment with arch length preservation strategies in the mandibular arch
Comparison	Untreated control group of children, children with treatment other than arch length preservation
Outcomes	Mandibular second permanent molar eruption disturbances as evidenced radiographically
Study design	Randomized control trials
	Prospective cohort studies
	Retrospective studies

This review considered the studies pertaining to arch length preservation utilizing lingual holding arch and lip bumper appliance as an interceptive procedure (non-extraction treatment). The study designs included randomized controlled trials (RCTs), prospective cohort studies, and retrospective studies. All the studies should have reported follow-ups before and after orthodontic evaluation. The review included all publications from different languages without any restriction. Exclusion included scripts from review papers, letters to editor, case reports, cases with extraction modality, multiple publications on same pool of patients, and animal studies on the review topic.

### Information sources and search

An electronic search was conducted in the following databases to identify the relevant studies: National library of Medicine (MEDLINE-PubMed) via PubMed, Cochrane Central Register of Controlled Trials, Cochrane’s Oral Health Group’s Trials Register, Clinicaltrials.gov, Google scholar, and other journal databases (Elsevier, Wiley, Oxford Academic, SAGE journals) from inception up to September 2020. A manual search of the reference source from all the selected full text articles and review articles on the subject identified relevant studies. Table 2 tabulates search strategy and key words.

**Table 2:** Search strategy

Database	PubMed	((((((((("E space preservation"[All Fields] OR "Leeway space"[All Fields]) OR "E space"[All Fields]) OR "arch length preservation"[All Fields]) OR "nance lingual arch"[All Fields]) OR "lingual arch"[All Fields]) OR "lingual holding arch"[All Fields]) OR "lip bumper"[All Fields]) OR "Schwarz appliance"[All Fields]) AND ("dentition, mixed"[MeSH Terms] OR ("dentition"[All Fields] AND "mixed"[All Fields]) OR "mixed dentition"[All Fields] OR ("mixed"[All Fields] AND "dentition"[All Fields]))) OR ("Mandibular second molar impaction"[All Fields] OR (("mandible"[MeSH Terms] OR "mandible"[All Fields] OR "mandibular"[All Fields]) AND second[All Fields] AND ("molar"[MeSH Terms] OR "molar"[All Fields]) AND ("tooth eruption"[MeSH Terms] OR ("tooth"[All Fields] AND "eruption"[All Fields]) OR "tooth eruption"[All Fields] AND difficulty[All Fields])) OR (("mandible"[MeSH Terms] OR "mandible"[All Fields] OR "mandibular"[All Fields]) AND second[All Fields] AND ("molar"[MeSH Terms] OR "molar"[All Fields]) AND ectopic[All Fields] AND "eruption"[All Fields]))
	Cochrane registry, CENTRAL Clinicaltrails.gov, Google scholar, journal database (Elsevier, Wiley, Oxford Academic, SAGE journals)	"Mixed dentition", “transitional dentition”, “Leeway space” "E space", "lingual holding arch", “Nance holding arch”, "lip bumper", "Schwarz appliance", "mandibular second molar impaction", “mandibular second molar eruption disturbances”, “mandibular second molar eruption difficulties”
Filters		None
Journals searched through journal database		European Journal of Orthodontics, Journal of Orthodontics, Journal of Clinical Orthodontics, Seminars in Orthodontics, American Journal of Orthodontics & Dentofacial Orthopedics, Angle Orthodontist, and Orthodontics & Craniofacial Research

### Study selection

Two independent reviewers (SA and IS) scrutinized titles and abstracts of the potentially qualifying studies. The reviewers conducted the assessment of the full texts independently for relevance. A third reviewer (JJ) resolved any disagreement between the first two reviewers by consensus.

### Data collection process and data items

Data were extracted by 2 reviewers independently from each included study and entered in an electronic spreadsheet that included the following information: name of the author, year of publication, study design, sample size, inclusion criteria, appliance type (arch length preservation strategy), appliance wear duration, treatment duration, outcome assessment, mean anterior mandibular crowding, M2 eruption problems, and percentage of eruption difficulty.

### Risk of bias (ROB) within studies

The quality assessment tool for observational cohort and cross-sectional studies assessed the selected studies independently [23]. This assessment tool contained 14 questions focusing on the assessment of the internal validity of the study. Each study was evaluated based on the information of the study design and execution and how well the confounding factors were handled to minimize bias. Accordingly, the tool accorded good, fair, or poor ratings to the studies. The Kappa (k) coefficient formalized the agreement between the reviewers with data extraction [24].

## Summary measures

Measurements for the outcome were based on nominal data which provides information about impaction or eruption difficulty from dental radiographs.

### Synthesis of results

Guidance from the Cochrane handbook of systematic review and RevMan 5.3 software (Review Manager, RevMan V.5.3, Copenhagen, The Nordic Cochrane Centre, The Cochrane Collaboration, 2014) assisted to perform the meta-analysis in a fixed-effects model [25]. Meta-analysis was performed for two studies with controls that employed lingual holding arch as a means of arch length preservation. The dichotomous data were presented as odds ratio and 95% confidence interval (CI). Cochran Q test along with I squared statistics estimated the heterogeneity. I squared statistics range from 0 to 100%. An I squared index less than 25% is indicative of low heterogeneity, between 75% -25% represents average heterogeneity, and more than 75% means that considerable heterogeneity is present [26].

### Risk of bias across studies

The quality of evidence of the outcome in the meta-analysis was evaluated using the Grading of Recommendations Assessment, Development, and Evaluation (GRADE) system. The following criteria were included for assessment of the quality of evidence for the outcome across studies: study design; ROB; consistency; precision; publication bias; and other considerations. Consistency was judged based on the heterogeneity (I squared) of the outcome and was ranked as: not serious—zero to 30%; serious—30 to 75%; and very serious—greater than 75 percent. Precision was judged based on the crossing of the CI of the pooled outcome to the no-effect line and the total sample size; it was ranked as “not serious” if total sample size was larger than 40, “serious” if between 20 and 40, and “very serious” if smaller than 20. Publication bias could be assessed when outcome had more than 10 articles included for quantitative analysis. The GRADE system results in four grades in rating the quality of evidence: (1) high; (2) moderate; (3) low; and (4) very low.

## Results

### Study selection

**Fig. 1:**
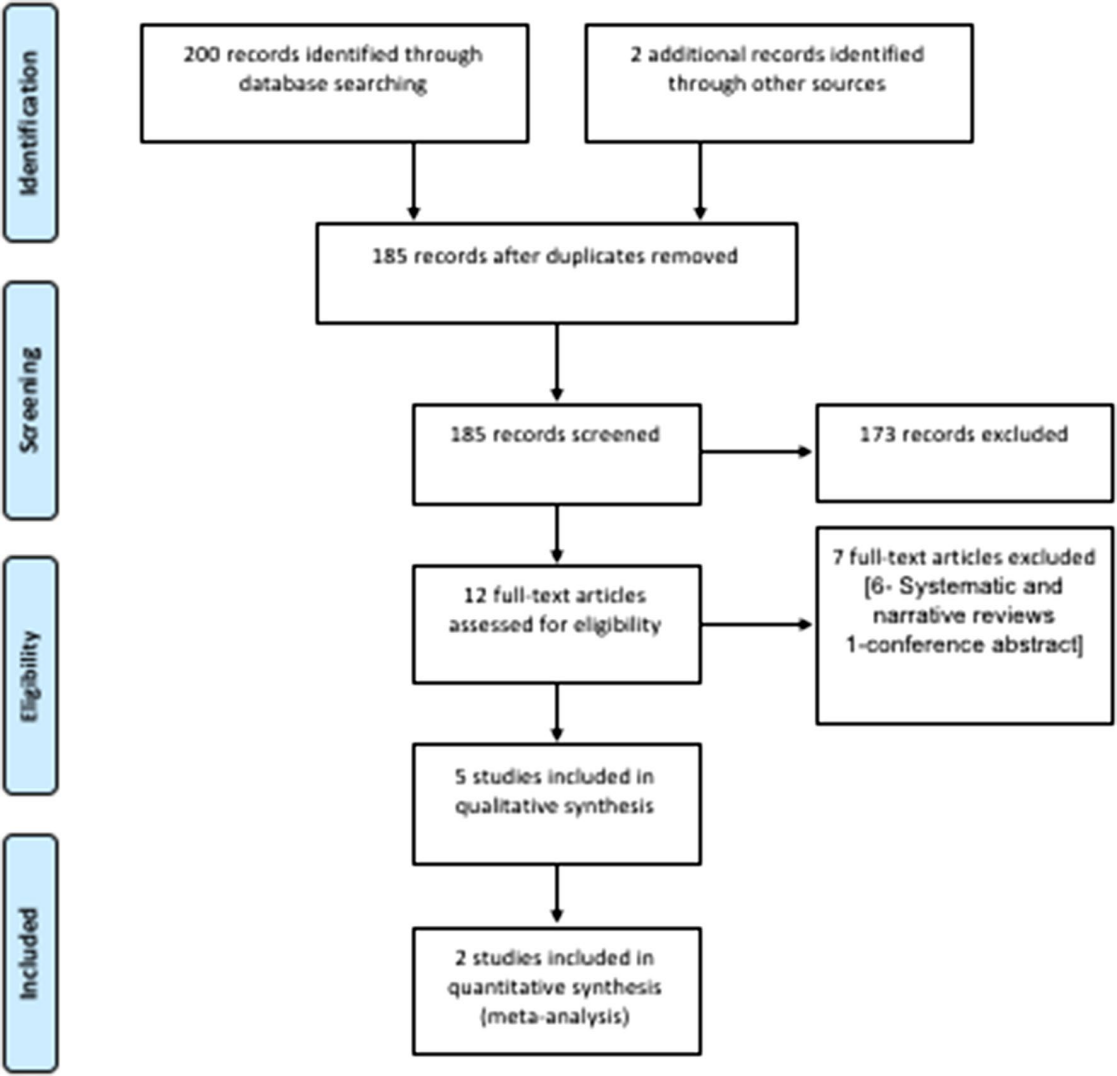
Flow diagram of study selection according to PRISMA statement

200 studies were obtained from electronic search and two records were identified through hand searching. After duplicates were removed, 185 records remained. Another 173 records were excluded after reading the titles and abstracts. A total of twelve records were found eligible for full text screening. Following full-text assessments, 7 articles were excluded: 6 articles were narrative or systematic reviews related to the use of similar appliances (lingual holding arch/lip bumper) but with different outcome measurements, and 1 article was a conference abstract on the topic. Finally, five studies were included in qualitative synthesis [15–19] and two studies were included for meta-analysis [17, 18] (Fig. 1). The Kappa statistic indicated “almost perfect” inter- examiner reviewer agreement (k = 0.91, 95% CI: 0.89 to 0.94).

### Study characteristics

Detailed descriptive data of the included studies are listed in Table 3. Of the five included studies, two were prospective cohort studies [16, 17] and the remaining three were retrospective studies [15, 18, 19]. Two studies used a lip bumper [15, 19] and three studies used a lingual holding arch [16–18]. The search did not identify any randomized controlled trials. The year of publication of the included studies ranged from 2011 to 2020. In total, 1222 participants were part of these studies, out of which 855 participants underwent arch length preservation for the relief of minor crowding and 367 participants were in the control group. Of the two lip bumper studies, only one reported full time wear (24 h per day) [15] and the other did not specify [19]. Also, it was reasonable to assume that the lingual holding arch is not removable and therefore would be worn full time [16–18]. The treatment duration ranged from 7 to 75 months.

**Table 3:** Characteristics of the included studies

Study	Study design	Study group	Untreated control group	Inclusion criteria	Appliance type (arch length preservation strategy)	Appliance wear duration	Treatment duration
Ferro et al. [15]	Observational, Retrospective cohort	N = 260 Mean age (years): 10.2	N = 135	Anterior mandibular crowding of 2 mm or more	Mandibular lip bumper—the gingival level in the vertical plane, under the action of the perioral muscles	24 h/day	28 months average (range, 7–75 months)
Jacob et al. [19]	Observational, Retrospective cohort	N = 67 Mean age (years): 10.6 ± 1.3	No control	Not specified	Mandibular lip bumper—Each LB was adjusted so that the acrylic shield was 2–3 mm away from the labial surface of lower incisors and 4–5 mm away from the facial surfaces of buccal segments	Not specified	0.8 to 2.9 years
Rubin et al. [17]	Prospective longitudinal study	Mandibular lingual holding arch (N = 85) Mean age (years): 9.5	N = 100 Mean age (years): 8.8	Mild to moderate crowding in the mandibular dental arch of 2–4.5 mm	Mandibular lingual holding arch (N = 85) The passive mandibular lingual holding arch was constructed with bands on the mandibular first permanent molars, with a soldered .036-inch stainless steel archwire extending lingually and anteriorly along the arch. Anteriorly, the archwire was fabricated to lie passively just below the cingula of the canines and the incisors	24 h/day	2.1–2.5 years
Sonis and Ackerman [16]	Prospective study	N = 200 Mean age (years): 11.2 (9.9–11.9)	Historic control	A non-extraction treatment approach with E-space preservation utilizing passive lingual holding arches	Passive lingual holding arches	Not specified	Not specified
Arevalo et al. [18]	Retrospective study	N = 126 Mean age (years): 8.78 ± 1.52	N = 132 Mean age (years): 9.49 ± 1.75	Lower lingual holding arch or a bilateral maxillary space maintainer and presence of good quality panoramic and/or periapical radiographs	Lower lingual holding arch	Not specified	Not specified

### Risk of bias within studies

Quality assessment of the included studies revealed that one study was good, three studies were fair, and another study was poor with moderate risk of bias (Table 4). Only two studies reported sample size calculation [17, 18], and this could implicate the lack of adequate size and effect in other studies. Statistical analysis to control the potential confounding variables which were not of interest were measured in four of the included studies [15–18].

**Table 4:** Quality assessment using quality assessment tool for observational cohort and cross-sectional studies

Criteria	Sonis and Ackerman [16]	Ferro et al. [15]	Rubin et al. [17]	Jacob et al. [19]	Arevalo et al. [18]
Was research question or objective in paper clearly stated?	√	√	√	√	√
Was study population clearly specified and defined?	√	√	√	√	√
Was participation rate of eligible persons at least 50%?	√	√	√	√	√
Were all participants selected or recruited from the same or similar populations (including the same time period)? Were inclusion and exclusion criteria for being in study prespecified and applied uniformly to all participants?	√	√	√	X	√
Was sample size justification, power description, or variance and effect estimates provided?	X	X	√	X	√
For analyses in this paper, were the exposure(s) of interest measured prior to the outcome(s) being measured?	√	√	√	√	√
Was timeframe sufficient so that one could reasonably expect to see association between exposure and outcome if it existed?	CD	√	√	√	CD
For exposures that can vary in amount or level, did study examine different levels of exposure as related to outcome (such as categories of exposure, or exposure measured as continuous variable)?	NA	NA	NA	NA	NA
Were exposure measures (independent variables) clearly defined, valid, reliable, and implemented consistently across all study participants?	√	√	√	X	√
Was exposure(s) assessed more than once over time?	NA	NA	NA	NA	NA
Were outcome measures (dependent variables) clearly defined, valid, reliable, and implemented consistently across all study participants?	√	√	√	√	√
Were outcome assessors blinded to the exposure status of participants?	NA	NA	NA	NA	NA
Was loss to follow-up after baseline 20% or less?	√	√	√	√	√
Were key potential confounding variables measured and adjusted statistically for their impact on relationship between exposure(s) and outcome(s)?	√	√	√	NR	√
Quality rating	Fair	Fair	Good	Poor	Fair

√ yes, *x* no, *CD* cannot determine, *NA* not applicable, *NR* not recorded

### Results of individual studies

The mechanism for arch length preservation varied between the strategies including harnessing the force from the lip during normal oral functions as in lip bumper or maintaining a passive support with lingual holding arch. Mandibular second molar eruption problems were noticed in lip bumper that ranged from 11.9 to 22% and passive lingual holding arch that varied between 4.7 and 14.5% [15–19]. These were primarily based on the status of eruption and the stage of root development, or position of the mesial cusps below the height of contour of the distal surface of the mandibular first molar (Table 5). However, one study failed to clearly describe the criteria for measuring the study outcome [19]. Of the 855 subjects, 130 subjects experienced M2 eruption problems with the difficulty of eruption ranging from 11.9 to 22%. The control group as reported by three studies, that demonstrated a prevalence of M2 eruption problems ranged from 1 to 2.96% [15, 17, 18]. One study did not provide details about the historic controls and hence, the control group details could not be considered in the analysis [16]. The nature of eruption difficulty included either impaction or ectopic eruption. A common predictor for the development of M2 impaction was the angulation greater than 24 degrees between first molar and M2 angulation concomitant with arch length preservation strategies [15, 16]. However, one study reported that a greater angulation could not be considered a significant predictor of M2 eruption difficulty [17]. With lip bumper protocol, distal tipping of the first molars or incorrect fitting of the first molar bands have been noted as the possible causes for M2 impaction [15, 19]. With lingual holding arch, either space-width ratio or molar angulation has been implicated as a predictor for M2 impaction [16, 17].

**Table 5:** Study outcome characteristics

	Measurement record	Outcome measurement	Age at outcome measurement	Mean mandibular anterior crowding	Mandibular second molar eruption problems	Other findings	Eruption difficulty
Ferro et al. [15]	Panoramic radiograph	Mandibular second molar considered erupted once it reached the functional occlusal plane with its mesial marginal ridge at the same level of the mandibular first molar’s distal marginal ridge	Treatment group (13.4 years)	Treatment group (3.8 mm; SD, 1.3)	Treatment group: 18 (impaction) + 41 (ectopic eruption), Total patients- 59 (22%)	9 patients showed bilateral impaction in treatment group; 1 patient showed bilateral impaction in untreated group	22%
		Impaction diagnosed for molars whose eruption was interrupted before gingival emergence by physical barrier or abnormal dental position and closed apices of the roots	Control group (13.8 years)	Control group (4.8 mm; SD, 2.1)	Control group: 2 (impaction) + 2 (ectopic eruption), Total patients- 4 (2.96%)		
Jacob et al. [19]	Panoramic radiograph	Not specified	Not specified	Not specified	Treatment group: 8 patients (11.9%)	Five (7.5%) patients showed unilateral second molar impaction while three (4.5%) showed bilateral impaction	11.9%
Rubin et al. [17]	Treatment group- Panoramic radiograph	No eruption difficulty when, with the root formed for 75% of its length or more, the second molar had erupted in the oral cavity	Treatment group (13.3 years)	2–4.5 mm	4.7% had eruption difficulty; 1% in the control group		Mandibular lingual holding arch- 4.7%
	Control group- Lateral oblique radiograph	Eruption difficulty, when the root of the mandibular second molar was at least 75% formed, but the tooth remained unerupted	Control group (12.8 years)				
Sonis and Ackerman [16]	Panoramic radiograph	Mandibular second molar impaction was defined as incomplete eruption when the distal cusps of the second molar were clinically visible and the mesial cusps were radiographically confirmed below the height of contour of the distal surface of the mandibular first molar	Not specified		29 patients had at least one impacted second molar (14.5%). [Twenty-four patients had unilateral impactions, and five were bilateral]		14.5%
Arevalo et al. [18]	Panoramic radiograph and/or periapical radiograph	Second molar impaction was defined as incomplete eruption when 75 percent of the roots had formed, and the distal cusp might be clinically visible but the mesial was radiographically confirmed below the height of contour of the distal surface of the mandibular first molar	Not specified but age controlled	Not specified	Treatment group: 18 teeth; 10 patients (impaction), 7.1%		7.1%
					Control group: 4 teeth; 2 patients (impaction), 1.5%		

### Synthesis of results

**Fig. 2:**
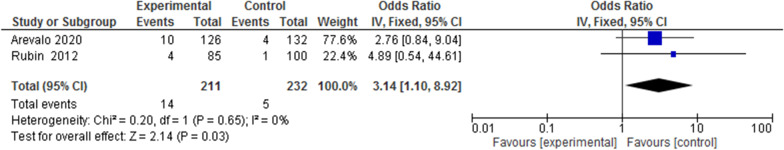
Meta-analysis odds ratio Forest plot [fixed effects] of included studies (95% confidence intervals)

**Fig. 3:**
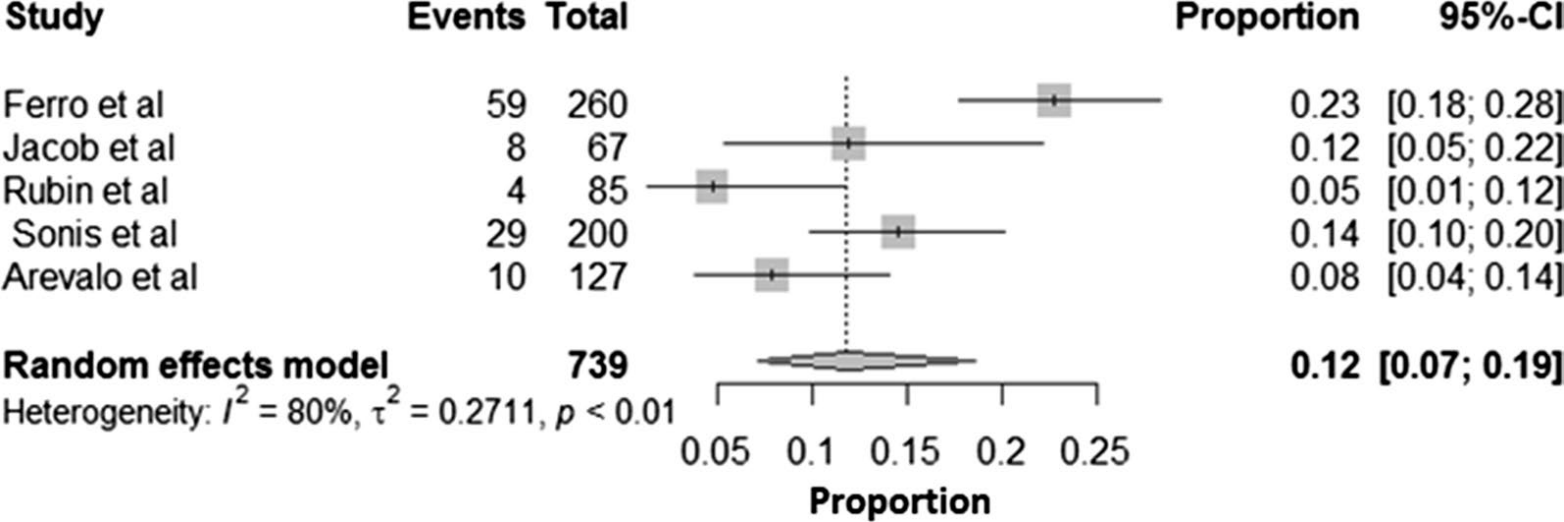
Proportion Forest plot [random effects] showing eruption difficulty for each study, plus pooled response (diamond) with 95% confidence intervals

Meta-analysis included two studies that employed lingual arch, and that reported M2 eruption difficulty ranging from 4.7 to 7.1% [17, 18]. The pooled data from the two studies revealed 3.14 times higher odds of developing M2 eruption difficulty at 95% CI (1.10–8.92). The studies observed no heterogeneity in the analysis (Fig. 2). Since there are only two studies contributing to the data for meta-analysis, we were unable to present the sensitivity analysis by excluding the studies. However, in addition to the analysis presented, we included a pooled estimation of intervention group percentage of difficulties in eruption grouping all studies using R software (Fig. 3). The aggregate eruption difficulty is around 12% based on five studies.

### Risk of bias across studies

The certainty of evidence was evaluated according to the GRADE approach. The bias elements were not downgraded in the grade approach. However, there is a serious problem: the imprecision domain in grade where the 95% CI is too wide to arrive at a precise conclusion. Funnel plots were not constructed as the data available for the meta-analysis is only two studies. For the outcome, mandibular second molar eruption disturbances, the certainty levels were graded as very low (Table 6).

**Table 6:** GRADE evidence profile [gradepro.org]

Outcomes	No of participants (studies) follow up	Quality of the evidence (GRADE)	Relative effect (95% CI)	Anticipated absolute effects	
				Risk with control	Risk difference with D (95% CI)
Mandibular second molar eruption disturbance	444 (2 studies)	⊕ ⊝ ⊝ ⊝	OR 5.2 (1.92 to 14.06)	Study population	
		VERY LOW^a^ due to imprecision		22 per 1000	81 more per 1000 (from 19 to 215 more)
				Moderate*	
				20 per 1000	76 more per 1000 (from 18 to 203 more)

GRADE Working Group grades of evidence

High quality: Further research is very unlikely to change our confidence in the estimate of effect

Moderate quality: Further research is likely to have an important impact on our confidence in the estimate of effect and may change the estimate

Low quality: Further research is very likely to have an important impact on our confidence in the estimate of effect and is likely to change the estimate

Very low quality: We are very uncertain about the estimate

*CI* confidence interval, *OR* odds ratio

^a^Indirectness: the 95% CI is too wide so, downgraded by one level

*The basis for the assumed risk (e.g. the median control group risk across studies) is provided in footnotes. The corresponding risk (and its 95% confidence interval) is based on the assumed risk in the comparison group and the relative effect of the intervention (and its 95% CI)

## Discussion

### Summary of evidence

This systematic review and meta-analysis explored and summarized the information associated with ALPS and potential M2 eruption difficulties, both ectopic eruption and impaction. There are two reviews on ALPS: one was a narrative review that studied the use of lip bumper and its subsequent effect on M2 eruption but included only case reports and other narrative reviews [27]. This narrative review reported M2 impaction in 7–12% of the treated group and 1.4% in the untreated group. The second was a systematic review that evaluated the effects of lip bumper therapy on the mandibular dental arch of children and adolescents as the primary outcome and M2 eruption disturbances as the secondary outcome [28]. A number of other studies reported first molar distalization or arch length changes with lip bumpers when compared with untreated controls [29–33]. A recent review reported that lingual arch did not increase the arch length significantly negating any change in the position of the mandibular first molars [8]. However, Viglianisi in another systematic review demonstrated 0.54° of first molar distal tipping with lingual arch [3]. Hence it is understandable, that the effect on the M2 eruption with lingual arch will be minimal. To avoid a possible bias, only lingual holding arch strategy was considered in the quantitative analysis. Till date, there is no systematic review or meta-analysis addressing lingual holding arch effects on the M2 eruption. Current meta-analysis revealed 3.14 times higher odds of developing mandibular M2 eruption difficulty with 95% CI (1.10–8.92) after arch length preservation modality with lingual holding arch. However, it is to be noted that the outcomes were pooled data of retrospective studies [15, 18, 19] and prospective cohort studies [16, 17].

The mandibular second molar eruption problems were noticed with both the active and passive strategies. In the active mode (lip bumper), it ranged from 11.9 to 22% and passive mode (lingual holding arch) demonstrated eruption problems ranged between 4.7 and 14.5%. The pooled estimate of eruption difficulties in the intervention group demonstrated a 12%. Given the influence of active nature of the appliance on the first molar, the lip bumpers not only maintained arch length, thus preserving leeway space, but (particularly if advanced) distalisation of the lower first molars took place [29, 34]. However, few investigations noted that the changes in the arch length happened irrespective of the second molar status [35, 36]. There are equivocal conclusions with regards to predictive factors for mandibular M2 in the literature [15–17]. For lingual arch therapy, Sonis and Ackerman reported an increased risk of M2 impaction when the inter-molar angulation exceeded 24° [16], but contradictory to Rubin et al. assessment where higher angulation is not a predictor [17]. First molar/M2 spacing, presence of third molar, space width ratio, facial pattern, skeletal relationship, gender, and age proved to be poor predictors of mandibular M2 eruption difficulty [14–17]. Arevalo et al. on lingual arch ALPS noted that there was 6.53 times greater chance of M2 impaction compared to controls after controlling for age [18]. For every increase in age by one year, there was an increase in the odds of M2 impaction by 1.25 times after controlling for the appliance [18].

Further, studies that utilized lip bumper noted that an initial anterior crowding of more than 4 mm was a risk factor for M2 eruption [15]. When the duration of lip bumper therapy was more than 2 years, the odds of developing M2 eruption disturbance became higher and an altered eruptive path was consequential [15]. Bergersen noted first molar distalization in 95% of the patients under lip bumper therapy with its increased duration of use coupled with the number of times it was linearly advanced [30]. Another study by Shapira et al. reported that the deficient mesial root length of the M2 as the primary impaction factor [37], but this notion was not analyzed in any of the primary studies in the present systematic review. More research is warranted to extrapolate if the effects on the second molar differ between the appliances (lip bumper/lingual arch).

Studies in the present systematic review measured the outcomes based on their own criteria and had the ages matched between the treatment and control groups [15, 17, 18]. But the definition of the criteria was not consistent across the studies. In one of the included studies, eruption difficulty was defined, when the root of the M2 was at least 75% formed, but the tooth remained unerupted [17]. In another study, closed apices of the roots irrespective of the 75% root completion was considered [15]. Another study did not give consideration to root development, instead defined impaction based on cuspal clinical visibility (Table 5) [16]. Another study noted 75% root completion along with distal cusp clinical visibility [18]. It could be extrapolated that there were no standard outcome measurement criteria employed in the literature.

## Limitations

The limitation of the present systematic review and meta-analysis was the extrapolation of evidence despite the lack of RCTs in this field of research. RCTs and prospective controlled trials are deemed necessary to provide a high-quality evidence. The primary studies that contributed to the review adopted no uniform criteria to measure the clinical outcomes.

## Conclusions

This systematic review demonstrates that ALPS pose a risk for development of mandibular second molar eruption disturbances, but the evidence was of very low quality. Methodologically sound prospective clinical trials are deemed necessary to provide higher levels of evidence.

### Implications for practice and future research

Within the limitations of this systematic review and meta-analysis, the authors intend to highlight a probable impending situation wherein a mandibular anterior discrepancy (crowding) was managed at the expense of creating a posterior discrepancy. The imminent consequence was the development of mandibular second molar eruption disturbances. The key implications are that preserving leeway space increases the risk of impaction of second molars. Further, the chances for development of posterior crowding needs to be considered in the treatment planning process.

Allen et al. reported a secular reduction in the mandibular leeway space in twenty-first century American White population and hypothesized that this reduction could influence the mesial migration of first molars and subsequent M2 eruption [38]. Future research is warranted to study the relationship between secular trends and mandibular leeway space in different races. This would allow for a thorough understanding of the underlying processes in the development of dental arch and help to establish clinical practice guidelines in the use of arch length preservation strategies. Further, with the lack of adequate evidence, the subject matter is a ‘hot topic’ for the researchers to conduct prospective trials. It is also recommended to perform multicentric studies to reduce the risk of performance bias in eventual RCTs.

## Data Availability

Date and materials are available on request from the corresponding author.

## Abbreviations

ALPS: Arch length preservation strategies
M2: Mandibular second permanent molar
PRISMA: Preferred reporting items for systematic reviews and meta-analyses
PROSPERO: International prospective register of systematic reviews
PECOS: Population, exposure, comparison, outcome, study design
RCTs: Randomized controlled trials
MEDLINE: Medical literature analysis and retrieval system online
ROB: Risk of bias
GRADE: Grading of recommendations assessment, development, and evaluation
CI: Confidence interval
